# Evaluation of Unobtrusive Microwave Sensors in Healthcare 4.0—Toward the Creation of Digital-Twin Model

**DOI:** 10.3390/s22218519

**Published:** 2022-11-05

**Authors:** Sagheer Khan, Imran M. Saied, Tharmalingam Ratnarajah, Tughrul Arslan

**Affiliations:** School of Engineering, The University of Edinburgh, Edinburgh EH9 3FF, UK

**Keywords:** digital twin, healthcare 4.0, Industry 4.0, unobtrusive sensors, microwave sensing

## Abstract

The prevalence of chronic diseases and the rapid rise in the aging population are some of the major challenges in our society. The utilization of the latest and unique technologies to provide fast, accurate, and economical ways to collect and process data is inevitable. Industry 4.0 (I4.0) is a trend toward automation and data exchange. The utilization of the same concept of I4.0 in healthcare is termed Healthcare 4.0 (H4.0). Digital Twin (DT) technology is an exciting and open research field in healthcare. DT can provide better healthcare in terms of improved patient monitoring, better disease diagnosis, the detection of falls in stroke patients, and the analysis of abnormalities in breathing patterns, and it is suitable for pre- and post-surgery routines to reduce surgery complications and improve recovery. Accurate data collection is not only important in medical diagnoses and procedures but also in the creation of healthcare DT models. Health-related data acquisition by unobtrusive microwave sensing is considered a cornerstone of health informatics. This paper presents the 3D modeling and analysis of unobtrusive microwave sensors in a digital care-home model. The sensor is studied for its performance and data-collection capability with regards to patients in care-home environments.

## 1. Introduction

The world has been transformed into an era of digitization and automation by Industry 4.0 (I4.0), or the fourth industrial revolution. This revolution has transformed not only digital industries, but also communication, healthcare, offices, and homes. It has touched every aspect of our lives. Furthermore, this change did not occur overnight, but took decades of research and experimentation. The term “fourth industrial revolution” was coined by Klaus Schwab in 2015, but the original industrial revolution started in 1850. This earlier revolution made use of the power of water and steam to mechanize production. Electrical power for mass production was utilized in the second revolution. The third was driven using electronics and information technologies to create automated production. The fourth revolution is the fusion of different technologies that blur the lines between the physical, digital, and biological spheres.

The I4.0 concept utilizes many technologies, such as big data [1,2], the Internet of Things (IoT) [3], artificial intelligence (AI) [4], and cyber-physical systems (CPS) [5] to achieve this automation or connectivity through continuous data sharing. This amalgamation has had an impact on society and is expected to continue further as new technologies are developed and implemented. The authors of [6,7,8,9] discussed the origin, examples, impact, and future trends of I4.0. In terms of industries, a smart factory stands for completely automated and connected machines, which can operate without the presence of humans by acquiring data, processing, and performing necessary actions [7]. The author of [10] created an illustration based on [11,12,13,14,15,16,17] for smart factories. Furthermore, the I4.0 concept is not limited to industries, but is applicable in other aspects of our lives, such as healthcare.

**Figure 1:**
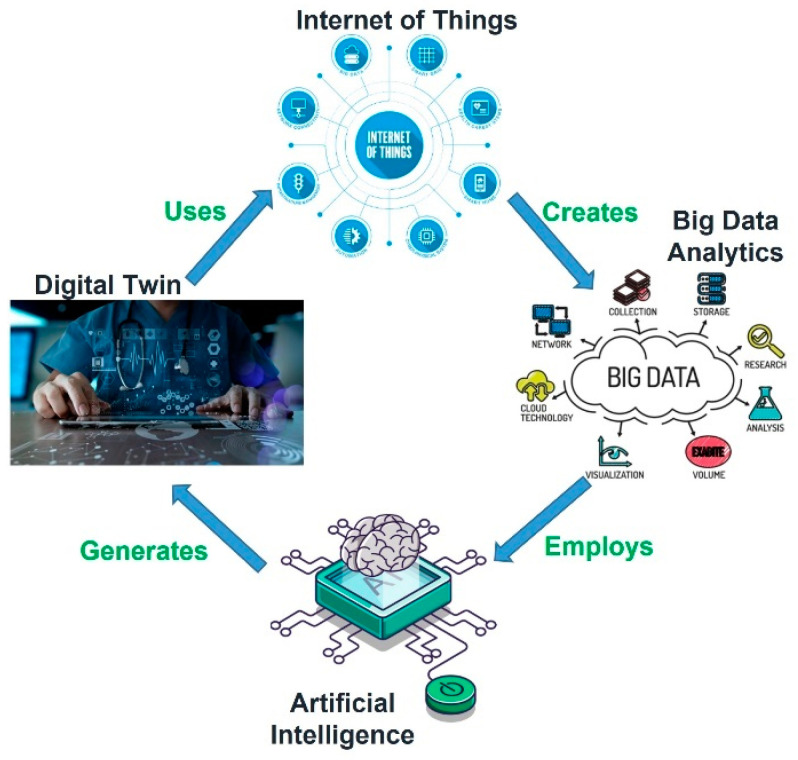
Technologies’ respective contributions toward creation of Digital Twin in healthcare.

The technologies of AI, big data, cloud computing, IoT, and CPS have made it possible to connect physical and virtual worlds. This cyber-physical integration involves a step toward the digitization of healthcare and industries [18]. The Digital Twin (DT) concept is based on the idea of representing the physical system in a digital model with all its properties and features. It focuses on bi-directional communication between the digital model and the physical world. The DT model of a system is made possible by the collaboration of multiple technologies, such as AI, ML, big data [19], IoT, and CPS. With real-time data provided by sensors, the DT model can perform many tasks, including analysis, comparisons, predictions, and simulations of a patient or system in healthcare and industries [10]. An extensive literature review of IoT and its associated technologies in healthcare is discussed in [20]. Figure 1 depicts the cycle of how multiple technologies contribute to creating a DT model.

The authors of [21,22] have studied different definitions of DT. NASA and Grieves gave two definitions that are accepted globally. NASA defines DT for a space vehicle as ‘‘an integrated multiphysics, multiscale, probabilistic simulation of an as-built vehicle or system that uses the best available physical models, sensor updates, fleet history, etc., to mirror the life of its corresponding flying twin”. [23] Originally, DT was regarded as a next-generation simulation tool [24] but the authors of [25] worked to create a convergence between the digital and the physical systems. DT models offer a better human–machine connection. Such digital representations of physical systems help in the monitoring of health, avoiding downtime/delays, predictive analysis, and improving product design with lower cost [26]. Over the years, numerous companies have utilized the DT concept. Siemens, for example, utilized DT to reduce the time to market, create new business directions, and minimize failures [27,28,29]. Similarly, Chevron saved millions of dollars in maintenance costs for its oil refineries with the help of DT [30].

Over the last few years, the increasing growth of the population has placed a massive burden on the current healthcare system and resources [31,32,33]. DT can ideally replicate the human body. It will use enormous data and AI-enabled models to reproduce human physiology and find solutions to various clinical questions [34]. The authors of [35] presented a summary of the technologies and requirements for the implementation of DT for personal healthcare. In [36], the importance and challenges of DT in personal healthcare are discussed. In Ref. [37], the authors elaborated on the implementation of DT in medicine, i.e., in medical CPS [38,39]. In Ref. [40], the authors provided a framework for DT in remote surgeries. A context-aware healthcare system using the DT framework was given in [41], which presented a rhythms classifier of Electrocardiogram (ECG) signals based on ML to detect heart abnormalities and diagnose diseases. An architecture for the DT of the heart, Cardio Twin, is utilized for ischemic-heart-disease detection [42]. Oklahoma State University’s Computational Biofluidics and Biomechanics Laboratory developed a DT model for the human airway system [43,44,45,46]. A DT model for hospital-management optimization is used by GE healthcare. It is centered on predictive analysis and AI capabilities to transform large amounts of data into actionable intelligence. John Hopkins Hospital in Baltimore has implemented the “Capacity Command Center” designed by GE Healthcare for decision-making and simulation capabilities. Siemens Healthineers has optimized Mater Private Hospital (MPH) in Dublin through DT technology. It was implemented in the radiology department with the help of AI models for the department and its operations. MPH overcomes the problems of increasingly old infrastructure, clinical complexity, increased patient demands, and the processing of big data through DT. The DT models have the advantage of predicting the outcomes of clinical procedures. This will help young doctors, surgeons, and practitioners to work in a protected environment to perform procedures and surgeries and analyze the behavior of the human body through the digital model. However, there are many privacy, technical, and ethical issues to be resolved before this can take place in practice.

Smart healthcare informs people about their current health status and allows them to manage some of their conditions by themselves. Technologies such as IoT, big data, AI, machine learning (ML), etc. can create automated and interactive systems. The utilization of the concept of I4.0 in healthcare is referred to as Healthcare 4.0 (H4.0) [47,48]. H4.0 helps in the provision of accurate medication, improved healthcare quality, preventing and managing emergencies, reducing healthcare costs, and early diagnosis of diseases [49,50]. H4.0 is one of many application areas that can benefit from the integration of DT technology. However, DT is a new field with open research, such as the utilization of better communication technologies, including 5G, 6G, and IEEE 802.11ah, or unobtrusive microwave sensing for improved communication, creating dynamic DT models, big-data analytics, the implementation of Edge, Fog, and Cloud within DT, and cyber security [10].

**Figure 2:**
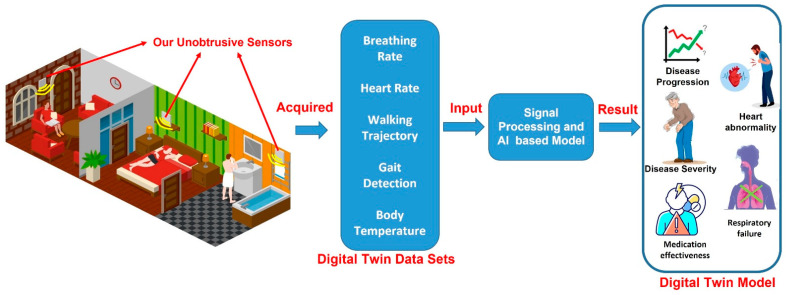
Proposed framework for creating DT model through the DT data sets collected through unobtrusive microwave sensors.

Figure 2 provides insights into the creation of a DT model with the help of DT datasets collected through unobtrusive sensors. The data collected through the unobtrusive sensors can be used in the creation of multiple DT models, especially for the elderly, in terms of breathing-rate data to monitor respiration failure and heart-rate data to monitor heart abnormalities. Walking trajectory and gait detection can also be recorded to determine the patient’s position in the room, the occurrence of falls, and the overall general behavior of the patient in the room. The data collected are provided as input to the signal processing for pre-processing, which is followed by the implementation of AI algorithms to extract and present the data in a useful manner. The authors of [51] provide a cloud-based framework of DT for elderly care. The authors performed the real-time supervision of a patient based on their normal and arrhythmic ECG-signal responses to multiple medications. The data acquired through sensors need to be pre-processed before use. The authors of [52] performed the non-contact monitoring of human respiration using infrared thermography and machine learning. The authors provided a comparison of multiple filters for data processing and the k-Nearest Neighbor (k-NN) classifier to differentiate between normal and abnormal breathing patterns. This work will help in the pre-processing and of normal and abnormal vital signs. Similar work was performed by the authors of [53].

To create a DT model, a considerable amount of data needs to be collected and processed. Accurate data collection is not only vital for the accurate diagnosis of disease but also the creation of a precise DT model. By utilizing real-time sensor data along with AI, ML, and big-data analytics, DT can be used for diagnostics, monitoring, prognostics, and optimization. The research aims to use microwave-sensing technology, unobtrusively, for data collection in a static care-home model created in the microwave-modeling tool of CST Studio Suite by Dassault Systèmes’. This study will help in the understanding of sensor placement, performance, and data-collection capability in a healthcare environment. This work will provide the foundation to create a DT model with realistic patient data, acquired through unobtrusive microwave sensing, for healthcare solutions with the help of AI, ML, and big-data analytics.

The paper is organized as follows. Section II describes the sensor design with a subsection elaborating on the sensor-performance analysis. Section III focuses on the information collected by 3D modeling and the analysis of static care-home models with multiple unobtrusive sensors.

## 2. Sensor Design

Sensors can be integrated into the living environment, clothing, and accessories in such a way that health-data acquisition can be conducted extensively and seamlessly. The main task of unobtrusive sensing is to allow the continuous monitoring of patients’ physical, physiological, and biological details in their daily lives without the technology impeding the day-to-day activity of the patient. Some of the most common vitals are ECG, ballistocardiogram (BCG), blood pressure (BP), blood-oxygen saturation (SpO_2_), heart rate, physical activities, and posture.

Unobtrusive sensing is a very suitable option for elderly patients in care homes or hospitals. Cameras and image-recognition software can also be considered forms of unobtrusive sensing; however, the use of cameras can create questions revolving around the privacy of the patient in their environment. Unobtrusive microwave sensing provides continuous monitoring of physical activities and behaviors, as well as physiological and biochemical parameters, during the daily life of the subject without any inference. The use of unobtrusive microwave sensors focusing on patients’ vital data, heart rate, breaths per minute, gait detection, and tumor detection/progression can raise no such questions of privacy violation. The data collected by the microwave sensors can provide more holistic information, which helps to create more detailed DT models. Unobtrusive sensors can be placed in two ways:

1. Sensors are worn by the patients, e.g., eyeglasses, earrings, shoes, gloves, clothing, or watches.
2. Sensors are placed on objects in the environment, e.g., car seats [54], chairs [55,56], and bathroom scales [57].

**Figure 3:**
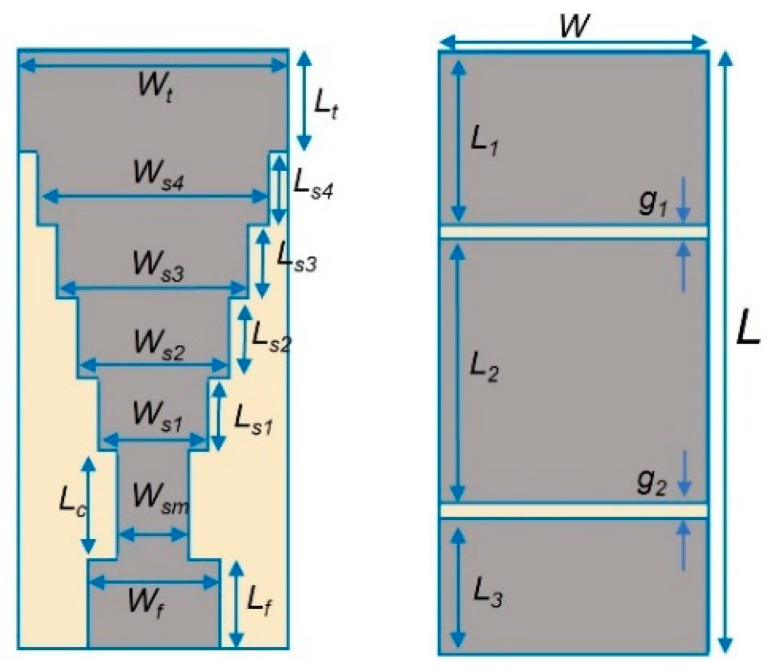
The geometry of the stepped monopole antenna.

**Figure 4:**
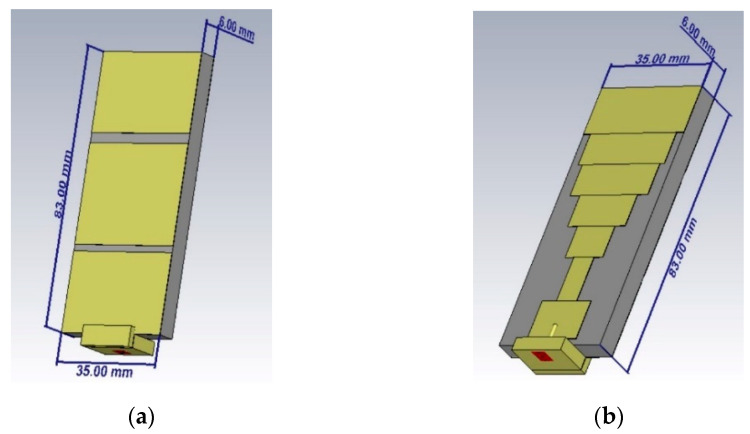
Stepped monopole antenna used as unobtrusive sensor (**a**) radiating front face (**b**) ground back face.

The authors of [58] utilized a stepped monopole antenna for its microwave unobtrusive sensing [59] ability to detect and create image zones of the brain that are affected by Alzheimer’s disease, providing an improvement over PET scans, which rely on biomarkers. The antenna was fabricated using conductive textile material (Shieldex Zell) and flexible substrate material (RS-PRO Viscose Wool Felt) to provide adaptability in the placement of the antenna [60]. The dielectric permittivity of the substrate (felt) was 1.55. The researchers were able to detect Alzheimer’s disease at an early stage of mild cognitive impairment. This research focuses on studying the sensor performance and data-acquisition capability in a care-home environment. The same sensor is intended to be utilized in future work for capturing realistic patient data to create a DT model for healthcare solutions. Figure 3 is the geometry of the stepped monopole antenna. Figure 4 represents the stepped monopole antenna with a frequency range of 0.5 GHz to 5 GHz designed in Dassault Systèmes’ CST Studio Suite. Table 1 provides details of the various parameters of the antenna.

Table 1 provides detailed parameter values of the stepped monopole antenna.

### Sensor Performance

**Figure 5:**
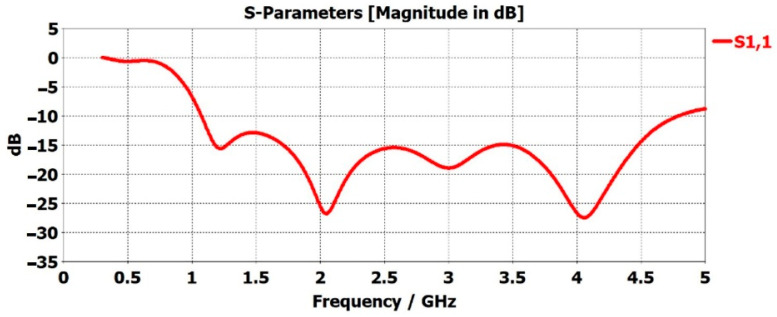
S-parameter, S_11_, of stepped monopole antenna in free space.

Figure 5 represents the scattering parameter (S-parameters) of the unobtrusive sensor in free space. S-parameters can be defined as the input wave that impinges on one of the ports. This is not an all-encompassing proposition. Some of the waves are reflected and others are transmitted. The quality of the reflected waves that are shown in the S_11_ plots, describes how many RF waves are absorbed or lost in the environment. This can be due to many factors, but in our work, it was found to have been due to the different situations of the subject in the care environment. In terms of propagation of the radio frequency (RF) waves, an antenna that crosses the −10-decibel mark is considered an operational antenna. The level of −10 dB is the gold standard by which an antenna is defined as operational in terms of propagating RF waves. If the antenna is designed in a way such that it crosses −10 dB at a certain frequency, this suggests that the antenna is functioning properly and can detect reflected RF waves, thereby receiving them.

**Figure 6:**
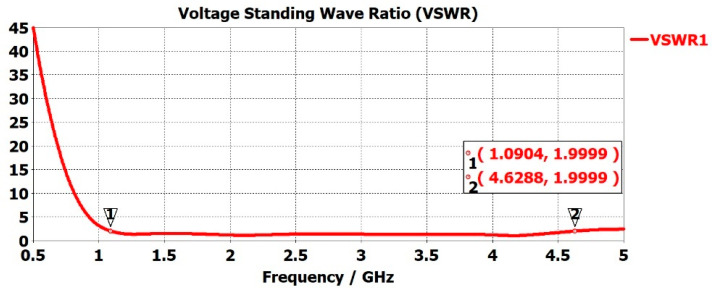
Sensor VSWR versus frequency response.

To deliver maximum power to the antenna, impedance matching needs to be conducted accurately. The Voltage Standing Wave Ratio (VSWR) is the measure of how well antenna impedance is matched. Low VSWR values mean the antenna is receiving maximum power and is well matched, but not all the power needs to be radiated. An antenna is defined as well matched when the VSWR value is below 2 [61,62,63]. Higher VSWR results in the reflection of more power at the antenna and, therefore, non-transmission. A large amount of reflected power at the antenna can damage the system. Figure 6 represents the VSWR of the sensor with the red curve. The VSWR was <2 for 1.0904 GHz < f < 4.6282 GHz.

## 3. Development of Care-Home Model in CST Studio

Over time, the increase in data from information and IoT sources is pushing hospitals, smart homes, industries, and smart cities to develop innovative models and tools. A large amount of data needs to be collected, stored, and processed to improve any system’s efficiency, scalability, and security. Smart-healthcare applications utilize big-data analytics, AI, and ML concepts for drug discovery, monitoring, intensive care, the training of healthcare professionals, and the diagnosis of diseases [64]. Neurodegenerative diseases, such as Alzheimer’s and Parkinson’s, respiratory-system failure, and stroke are very critical diseases and need to be detected as early as possible to provide a quick medical response. The currently used sensing and detection technologies have demonstrated progress in disease detection, but their large size, cost, and invasive nature remain drawbacks. Therefore, new sensing technology, which can be non-invasive, economically friendly, and small, needs to be tested. The flexibility of the sensors can be an advantage as this allows them to be placed on non-flat surfaces.

**Figure 7:**
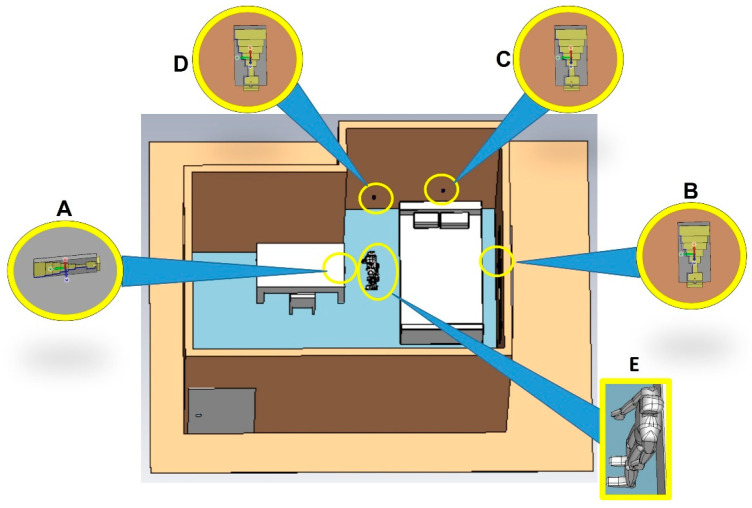
Digital model of the care home with unobtrusive sensor (**A**) on side of the table, (**B**) on the wall to the right, (**C**) above the bed, and (**D**) to the left of the bed; (**E**) electromagnetic human/patient model.

**Figure 8:**
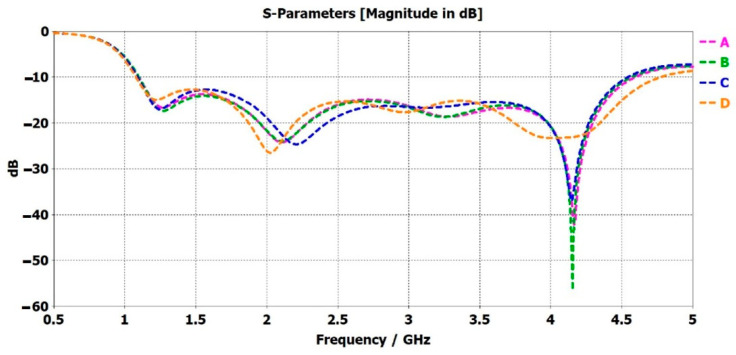
S-parameter (S_11_) in the model for the unobtrusive sensor (A) on side of the table (B), on the wall to the right (C), above the bed, and (D) to the left of the bed.

Dassault Systèmes’ CST Studio Suite was utilized to create the digital model for a care-home scenario with unobtrusive microwave sensors placed at different positions. Figure 7 represents the digital model, created in SolidWorks, and imported into CST Studio. This allowed the analysis of the simulated performance of the sensors. The properties of every item in the digital model are given in Solidworks. The human/patient model was given the properties of skin, the table was given wood, and so on, for the rest of the items in the model. The objects have different dielectric properties, and they behave differently when placed in front of RF waves. The S-parameters depend not only on the body’s dielectric properties but also on the patient’s position and standing/sitting direction. If a person is sitting and then sleeping in a similar experimental environment in front of the sensor, a different S-parameter response is observed. Hence, the position/placement of the antenna is important to detect the presence of the patient. The sensor is small and is not visible directly. A magnified version of the sensors is presented in Figure 7. The coordinate axes, i.e., U, V, and W, were placed in the magnified version. The direction of the W axis represents the radiating face of the sensor, which is directed toward the human or patient in the CST model. Figure 8 represents the S-parameters of the unobtrusive sensor placed at different positions (A, B, C, and D respectively) in the CST model. These figures had considerable power loss at certain frequencies compared to Figure 5. This shows that the human/patient presence in the CST model was detected by the sensor. The human body has different dielectric properties compared to the other objects in the CST model. The RF waves from the unobtrusive sensor that reach the patient and are absorbed more than the other items in the CST model. Hence, the placement of the sensor is correct and can be used to read the patients’ vital signs or detect diseases.

The s-parameter of the unobtrusive sensor, placed on the side of the table, is presented in curve A of Figure 8. The S_11_ showed a considerable loss of −42 dB at a frequency of 4.1 GHz. The sensor was in the direct line of sight of the patient. Due to the dielectric property of the human body, most of the RF waves were lost or were not reflected and received at the sensor. Compared to Figure 5, there was no such loss of RF waves. This shows that the sensor placement is accurate as it allows RF waves access to the patient’s body. The continuous data collected with the unobtrusive sensor in this position are significant and very beneficial for creating a DT model for reading vital signs or detecting diseases or respiratory failure in patient. Curve B in Figure 8, the sensor placed on the wall to the right, depicts a loss of −56 dB at 4.1 GHz, which is −14 dB more than curve A. The patient is more exposed to the RF waves, creating more opportunities for data collection.

Curves C and D in Figure 8 provide a power loss of −37 dB and −26 dB at 4.1 GHz and 2 GHz, respectively. These power-loss values were lower than the power-loss values in curves A and B in Figure 8. The sensor, placed above the bed, for curve C was not in the direct line of sight of the patient. The RF waves will have had very limited access to the human body. Reading the vital signs or detecting diseases or respiratory failure would have been very difficult. However, if the patient had been on the bed, the sensor would have had more access. Therefore, the placement of the sensor is an important factor in unobtrusive sensing. In terms of the s-parameter, the greater degree of loss in S_11_ means the patient was exposed more to the RF waves, suggesting a higher probability of collecting accurate data for healthcare. The Curve-D sensor was kept in the patient’s line of sight but intentionally not at the same level as that of the human body. It was placed at a lower position on the wall, close to the floor, and aimed at the feet of the patient. It provided a similar S_11_ performance to that of the free space in Figure 5, as it did not detect the presence of the patient. This sensor position was not suitable for data collection from the patient. However, if the patient had fallen on the floor, the sensor would have had the opportunity to detect the patient, gather data, and inform the medical personnel of the emergency.

**Figure 9:**
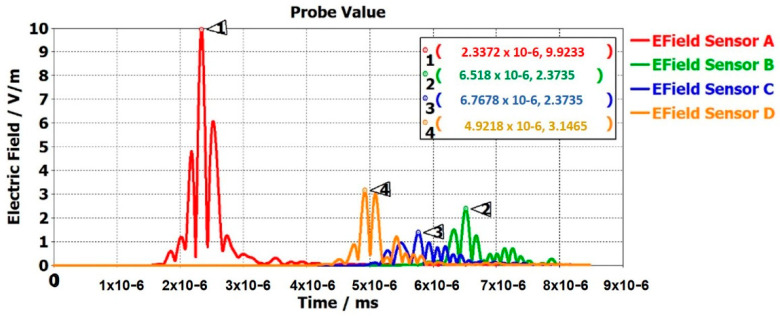
Efield of multi-sensor static digital model in CST Studio.

From the electrical signal of the antenna, the antenna performance and the general position of the patient can be known. An electrical field probe was placed at the head of the patient in the model to detect whether the electrical signal reached the probe and the time taken by the electrical signals from the antenna to the probe. Figure 9 shows that all the sensor’s electrical signals experienced different intensities and time delays based on their location. The sensor placed at position A took the shortest time, 2.33272 × 10^6^ ms to reach the probe of all the sensors. Sensor A was the closest to the human model; hence, it displayed the highest electric-field peak value, of 9.9233. This shows the sensors were working accurately. According to the CST model in Figure 7, if the patient moved closer to a other sensor, the highest electric-field value was achieved at that sensor. Therefore, the general location of the patient can be known if only the electric-field graph is studied with the knowledge of the sensor’s location. 

Table 2 provides the DT data sets collected from the 3D microwave modeling of the unobtrusive sensors. These DT data sets, along with the patient vitals acquired in future work with unobtrusive microwave sensors, will be pre-processed. AI–ML algorithms will be applied to the processed data to predict and classify various vital statuses as normal or abnormal and represent them in terms of a DT model. The total number of S_11_ values was 1002, over a frequency range from 0.5 GHz to 5 GHz; therefore, writing them into a table was not feasible. Figure 8 represents these values in the form of a graph. All the sensors were placed at different heights from the floor. The sensors were placed at variable distances and directions in relation the patient to test them from various positions. Table 2 provides various DT data sets to use for the creation of the DT model.

## 4. Conclusions

Collecting patient data unobtrusively and using them to create a Digital Twin (DT) model has huge research and practical potential. Unobtrusive microwave sensing is a unique way of reading patients’ vital signs or detecting diseases or gait changes from a distance. An unobtrusive microwave sensor was analyzed for its performance and its ability to collect DT data sets in a static care-home model. The analysis showed that the position of the sensors is important to sense the presence of and collect vital data from patients. Correct data collection is important for patient diagnosis and the creation of accurate DT models. The aim of future work will be to utilize the realistic patient DT data collected through unobtrusive microwave sensors with pre-processing and AI—ML algorithms for the creation of a DT model for the autonomous detection of patients’ vital signs, tumors, diseases, and gait.

## Figures and Tables

**Table 1:** Antenna-design parameters.

Parameters	Symbol	Value (mm)
Length	L	85
Width	W	35
Feeding Line L	L_f_	12
Feeding Line W	W_f_	17
QWTL Length	L_c_	14
QWTL Width	W_sm_	7
Step 1 Radiation-Patch Length	L_s1_	10
Step 1 Radiation-Patch Width	W_s1_	13
Step 2 Radiation-Patch Length	L_s2_	10
Step 2 Radiation-Patch Width	W_s2_	20
Step 3 Radiation-Patch Length	L_s3_	10
Step 3 Radiation-Patch Width	W_s3_	27
Step 4 RadiationPatch Length	L_s4_	10
Step 4 Radiation-Patch Width	W_s4_	30
Top Radiating-Patch Length	L_t_	15
Top Radiating-Patch Width	W_t_	35
Top Ground Place	L_1_	25
Middle Ground Plane Length	L_2_	30
Bottom Ground Plane Length	L_3_	25
Gap 1	g_1_	3
Gap 2	g_2_	2
Substrate thickness	h	6
Dielectric Permittivity of Substrate (Felt)	*ε* *_R_*	1.55

**Table 2:** DT-model data set.

Parameter	Sensor A	Sensor B	Sensor C	Sensor D
Height from floor	0.400 m	0.620 m	0.598 m	0.196 m
S-parameter (S_11_ dB)	−0.38, −0.39, −0.400, …., −7.71, −7.73, −7.73	−0.38, −0.39, −0.40, …, −7.68, −7.68, −7.58	−0.38, −0.39, −0.40, …, −7.29, −7.28, −7.29	−0.37, −0.37, −0.38, …, −8.66, −8.65, −8.63
Antenna-to-patient distance	0.359 m	1.562 m	1.428 m	0.958 m
Efield strength	9.9233 V/m	2.3735 V/m	1.3716 V/m	3.1465 V/m
Time to reach the E-probe	2.3372 × 10^−6^ s	6.518 × 10^−6^ s	5.7678 × 10^−6^ s	4.9218 × 10^−6^ s
